# Compound Mutations of the COL4A3 including a Novel Allele Identified in a Patient with Alport Syndrome

**DOI:** 10.1155/2020/1626378

**Published:** 2020-11-11

**Authors:** Zhendong Wang, Baichun Jiang, Shiqi Jin, Zhao Hu, Guangyi Liu

**Affiliations:** ^1^Department of Nephrology, Qilu Hospital, Cheeloo College of Medicine, Shandong University, Jinan, Shandong 250012, China; ^2^Key Laboratory of Experimental Teratology, Ministry of Education, Department of Genetics, School of Basic Medical Sciences, Cheeloo College of Medicine, Shandong University, Jinan, Shandong 250012, China

## Abstract

Alport syndrome (AS) is a hereditary nephropathy which is characterized by molecular abnormalities in collagen IV. Here, we report compound mutations of the COL4A3 gene including a novel allele identified in a patient with Alport syndrome. The patient was a 25-year-old Chinese woman. She has a history of proteinuria and hematuria with cleft lip and palate. The pathologic results were consistent with Alport syndrome. The patient received ACEI treatment but did not respond well to the treatment. Sequencing results revealed that the patient carried two heterozygous mutations in the COL4A3 gene, including a known mutation (c.4243G>C, p.G1415R), which was inherited from her father, and a previously undescribed allele (c.4216G>A, p.G1406R) inherited from her mother. To date, at least 294 different variants of COL4A3 have been reported according to the Human Gene Mutation Database (HGMD). Identification of c.4216G>A as a new AS-related mutation may contribute to both genetic diagnosis of AS and further functional study of COL4A3.

## 1. Introduction

Alport syndrome (AS) is a familial hereditary nephropathy caused by mutations in the type IV collagen genes COL4A3, COL4A4, and COL4A5 [1]. These genes encode *α*3, *α*4, and *α*5 collagen chains, which are the major structural components of the glomerular basement membrane as well as the basement membranes in the eye and cochlea. There are three types of heredity of Alport syndrome. The X-linked dominant inheritance of mutations in the COL4A5 gene accounts for 80%~85% of AS. The autosomal recessive inheritance of COL4A3 or COL4A4 mutations accounts for 10%~15% of AS. Dominant mutations in COL4A3 and COL4A4 have only been reported in about 5% cases [2–5]. High-throughput next-generation sequencing (NGS) technology is an efficient and appropriate form of genetic test for Alport syndrome, and more gene mutations are being discovered [6, 7]. Although the relationship between genotypes and phenotypes has not been fully understood in Alport syndrome, more discovery of genetic mutations would contribute to genetic diagnosis and counseling of Alport syndrome.

## 2. Materials and Methods

### 2.1. Case Report

The proband is a 25-year-old woman with proteinuria and hematuria. She has had otitis media for more than 20 years and had a cleft lip and palate operation when she was 5 years old. She went to the hospital for hematuria over the past year. No one else in her family suffers from proteinuria, hematuria, or renal hypofunction or deafness. Urine analysis showed 3+ proteinuria and 3+ hematuria (77 red blood cells per high-power field) and a urinary protein/creatinine ratio (P/Cr) of 4.56 g/g Cr. Albumin level was 32.5 g/L, and antinuclear antibodies were negative. She had renal biopsies. Recently, she took perindopril medicine by mouth.

Informed written consent was obtained from all participants. The study was approved by the Research Ethics Committee of the Qilu Hospital of Shandong University.

### 2.2. Methods

#### 2.2.1. Whole-Exome Sequencing

Peripheral blood was collected from EDTA anticoagulant vessels, and blood DNA was extracted with the extraction kit. DNA libraries were constructed with KAPA Library Preparation Kit. The vacuum-enriched DNA library samples were hybridized and captured in SureSelect hybridized buffer liquid series. Illumina NovaSeq high-throughput sequencing was performed on the captured DNA samples. The sequenced data were evaluated by Illumina Sequence Control Software (SCS), and bioinformatics analysis was performed. The data interpretation rules are based on guidelines from the American College of Medical Genetics and Genomics (ACMG) [8]. Variables named according to the rules of HGVS (http://www.hgvs.org/mutnomen) are given.

#### 2.2.2. Bioinformatics Analysis

To predict the pathogenesis of Col4A3 mutation, we use Mutation Taster software (http://www.mutationtaster.org/). This program can be used to predict whether the mutation is pathogenic or harmless. Similarly, Col4A3 amino acid sequences were analyzed using PolyPhen-2 (http://genetics.bwh.harvard.edu/pph2/) and SIFT software (http://sift.jcvi.org/). Based on straightforward physical and comparative considerations, the possible effects of amino acid substitution on the structure and function of human proteins were predicted by PolyPhen-2. Similarly, whether the protein function is affected can be predicted by SIFT. We checked the conservation status of this exonic variant, namely, Clustal X software in 6 species including a mouse, pig, sheep, rabbit, human, and pan.

#### 2.2.3. Sanger Sequencing

All the filtered mutations of this family were validated by Sanger sequencing. Specific primers were used to amplify the regional DNA fragments at the mutant sites. The sequences of the polymerase chain reaction (PCR) products were determined using the ABI 3730XL DNA Genetic Analyzer.

## 3. Results

### 3.1. Renal Biopsies

**Figure 1:**
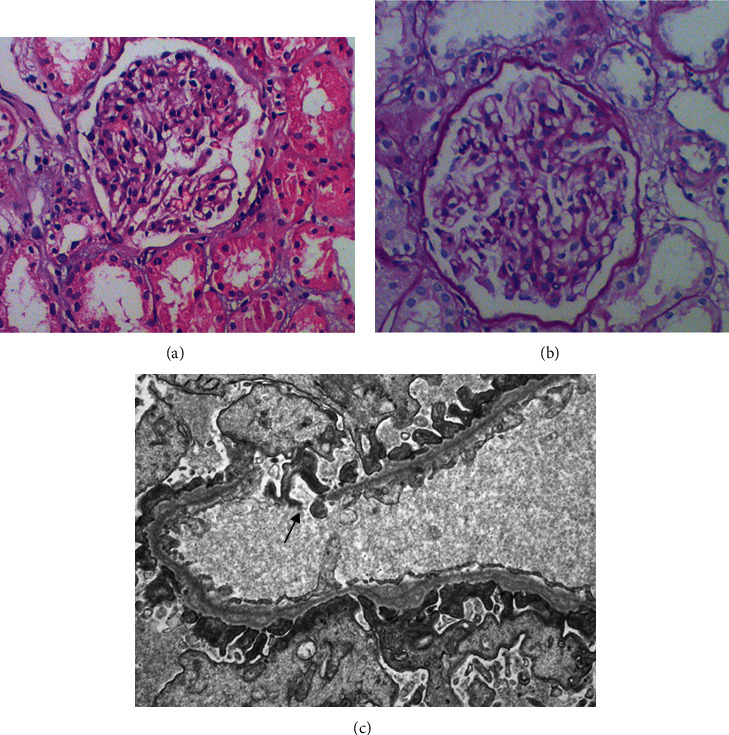
Light microscopy (a, b) and electron microscopy (c) of the patient's renal biopsy. One glomerulus showed mild mesangial hyperplasia, thickened glomerular capillary walls, and swelled podocytes. Electron microscopy demonstrated markedly irregular subendothelial GBM surface with splitting and “basket weaving” (black arrow).

The renal biopsies of patient are shown in Figures 1(a) and 1(b). We did H&E and PAS staining on the patient's renal biopsies. Electron microscopy (EM) was also performed. One out of the six examined glomeruli showed mild mesangial hyperplasia, thickened glomerular capillary walls, and swelling podocytes. EM demonstrated markedly irregular subendothelial GBM surface with splitting and “basket weaving” (Figure 1(c)).

### 3.2. Whole-Exome Sequencing

We detected two mutated sites in the COL4A3 gene (Table 1). The first one is a missense mutation c.4243G>C in the COL4A3 gene, resulting in amino acid change p.G1415R (glycine > arginine), which has been reported as a pathogenic mutation [9]. The other one is an undescribed missense mutation c.4216G>A, leading to replacement of the amino acid G1406 to R.

### 3.3. Bioinformatics Analysis

**Figure 2:**
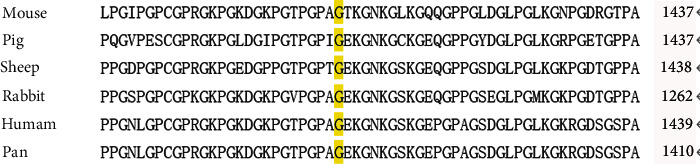
Glycine 1406 is conserved in collagen alpha-3 (IV) chain across different mammalian species.

Mutation Taster software analysis revealed that COL4A3 c.4216G>A (p.G1406R) mutation could be a disease-causing mutation with a score of 0.810. Also, SIFT software analysis revealed that COL4A3 c.4216G>A (p.G1406R) mutation could be a damaging mutation with a score of 0.912. Analysis with the PolyPhen-2 software mutation supports the probably damaging role of this amino acid change with a high score of 0.970. Clustal X software showed that the novel exon variant COL4A3 c. 4216G>A (p.G1406R) was conservative in primates (Figure 2), suggesting the possible pathogenicity of this exon variant in human beings.

### 3.4. Sanger Sequencing

**Figure 3:**
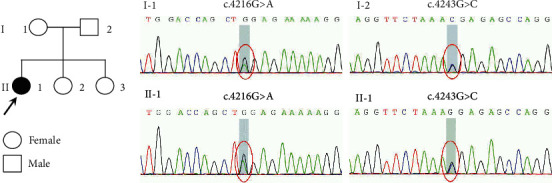
Pedigree and Sanger sequencing results of the patient's family.

The father and two sisters carry COL4A3 c.4243G>C mutation, and the mother is a carrier of COL4A3 c.4216G>A mutation. Since the parents and sisters do not have any symptoms, we conclude that the genetic mode is autosomal recessive inheritance (Figure 3).

## 4. Discussion

Although the patient does not have a family history of hematuria and chronic renal failure and no eye lesions in this study, she had hematuria and albuminuria, which gradually progressed to renal dysfunction. In EM, the basement membrane was segmentally thickened, shrunken, and broken, which is the clinical diagnostic criteria of Alport syndrome, according to Fliter [10] and Gregory [11]. Through clinical manifestation, family history, and renal pathological examination, Alport syndrome was highly considered.

In order to further clarify the diagnosis, we sequenced the patients with high-throughput next-generation sequencing. The results showed that there were two heterozygous mutations in the COL4A3 gene, c.4243G>C (p. G1415R) and c. 4216G>A (p.G1406R), which may cause the disease. The COL4A3 c.4216G>A (p.G1406R) mutation has not been reported and linked to Alport syndrome. Another mutation c.4217G>A (p.G1406E), which leads to amino acid change in the same glycine as c.4216G>A, was identified from two Japanese families [12], indicating this glycine may be critical for the protein function. The COL4A3 gene encodes a chain of 1670 amino acids. Missense mutations account for about 45% of COL4A3 mutations, among which about 85% are glycine substitutions in the conserved Gly-X-Y repeat sequence in the collagen domain of *α*3 (IV collagen) chain [4]. Glycine is the only amino acid without side chain and small enough to fit into the center of the triple helix structure. Substitutions of glycine in COL4A3 p.G3499A [13], p.G1406E [12], and p.G4235T [14] have been reported as pathogenic mutations. In this study, the novel missense mutation c.4216G>A causes substitution p.G1406R. Because arginine is larger and more polar than glycine, this substitution may reduce the stability of the complex and impair correct alignment of the type IV collagen triple helix [13, 15–17], leading to the decline of basement membrane filtration barrier function, which in turn causes Alport syndrome [13, 15]. The clinical symptoms are consistent with this prediction. In this patient, the basement membrane of the glomerular capillary wall was slightly thickened, shrunken, and broken. The segmental changes were reticulated in EM.

It is reported that 90% of men and 12% of women with XLAS develop end-stage kidney failure at the age of 40 [18]. ARAS is more serious and progresses faster than XLAS, and the median age of patients with renal failure is 21 years old [19]. This patient has mild symptoms (slight glomerular lesions and no renal failure) which may be related to the younger age of the patient or the shorter time of onset or that the mutation of the gene locus itself is less pathogenic and has little effect on the renal function of the patient.

Alport syndrome is often accompanied by hematuria, sensorineural deafness, and ocular abnormalities [20]. No abnormality in the eye, blood system, and heart ultrasound was observed in this case. The patient had congenital cleft lip. So far, there are no report suggesting mutation in COL4A3 may cause cleft lip and palate. The relationship between cleft lip and palate and COL4A3 gene needs further study.

## 5. Conclusion

In conclusion, via whole-exome sequencing in the combination of bioinformatics analysis strategy, we have identified a novel COL4A3 mutation (c. 4216G>A p.G1406R) in a suspected AS patient from China. Our study may expand the spectrum of COL4A3 mutations and contribute to genetic diagnosis and counseling of Alport syndrome.

## Figures and Tables

**Table 1:** Detected mutations.

Gene	Nucleotide changes	Amino acid change	Heterozygosity	SIFT	Polyphen-2	Mutation taste
Col4A3	c.4243G>C	p.G1415R	Het	Damaging	Probably damaging	Damaging
Col4A3	c.4216G>A	p.G1406R	Het	Damaging	Probably damaging	Damaging

## Data Availability

The data used to support the findings of this study are available from the corresponding author upon request.
